# Supplementary material for: Genomics of natural populations: gene conversion events reveal selected genes within the inversions of Drosophila pseudoobscura
Source: G3 (Bethesda). 2024 Jul 29;14(10):jkae176. doi: 10.1093/g3journal/jkae176 (PMC11457094; doi:10.1093/g3journal/jkae176)
Supplement: jkae176_Supplementary_Data [file jkae176_supplementary_data.zip › Figure_S2_G3-2024-405095.pdf]

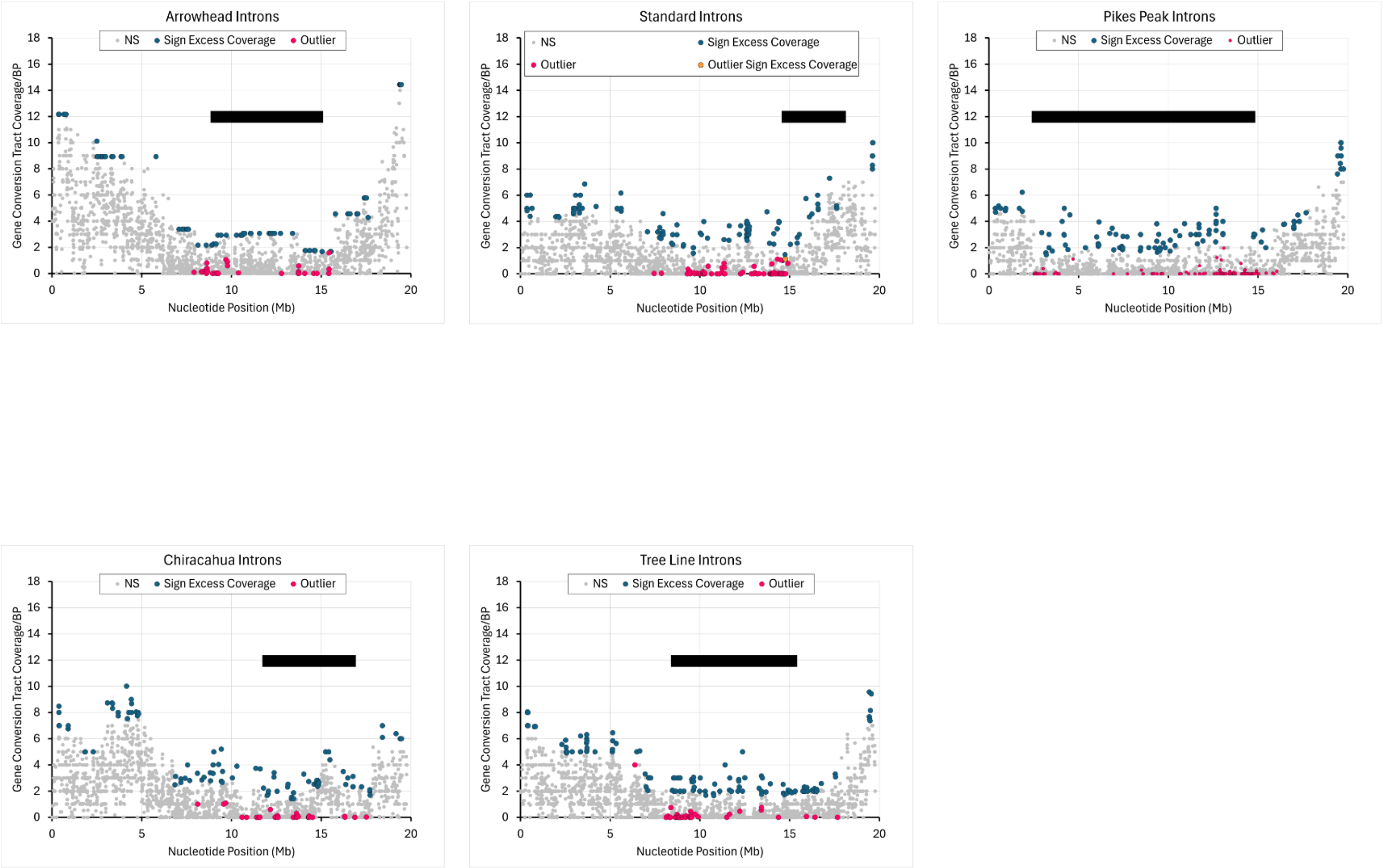

Figure S2. Mean gene conversion tract coverage in introns across Muller C in five gene arrangements in *D. pseudoobscura*. Coverage is the number of times that a nucleotide is covered by a gene conversion tract. Coverage was estimated for each intron within a transcript and the mean coverage for the entire transcript was averaged over all introns. Introns of outlier genes are shown with a pink marker while non-outliers are shown with a gray marker. The location of the derived inversion giving rise to each gene arrangement are shown with a black bar. Introns with excess coverage > 2SD are indicated.
