# Supplementary material for: Genomics of natural populations: gene conversion events reveal selected genes within the inversions of Drosophila pseudoobscura
Source: G3 (Bethesda). 2024 Jul 29;14(10):jkae176. doi: 10.1093/g3journal/jkae176 (PMC11457094; doi:10.1093/g3journal/jkae176)
Supplement: jkae176_Supplementary_Data [file jkae176_supplementary_data.zip › Figure_S3_G3-2024-405095.pdf]

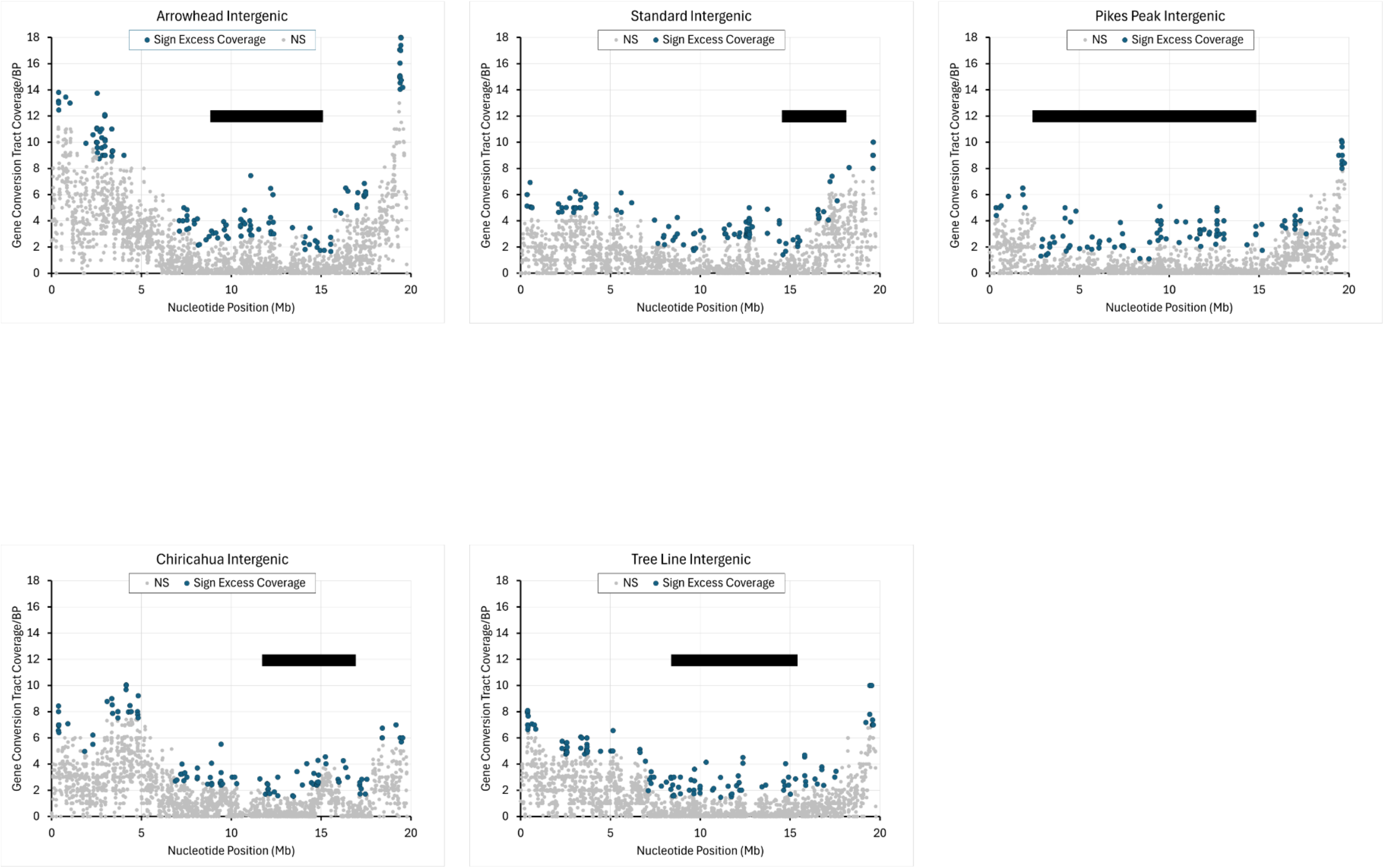

Figure S3. Mean gene conversion tract coverage in intergenic regions across Muller C in *D. pseudoobscura* in five gene arrangements. Coverage is the number of times that a nucleotide is covered by a gene conversion tract. The location of the derived inversion giving rise to each gene arrangement are shown with a black bar. Intergenic regions with excess coverage > 2SD are indicated.
