# Supplementary material for: Genomics of natural populations: gene conversion events reveal selected genes within the inversions of Drosophila pseudoobscura
Source: G3 (Bethesda). 2024 Jul 29;14(10):jkae176. doi: 10.1093/g3journal/jkae176 (PMC11457094; doi:10.1093/g3journal/jkae176)
Supplement: jkae176_Supplementary_Data [file jkae176_supplementary_data.zip › Figure_S4_G3-2024-405095.pdf]

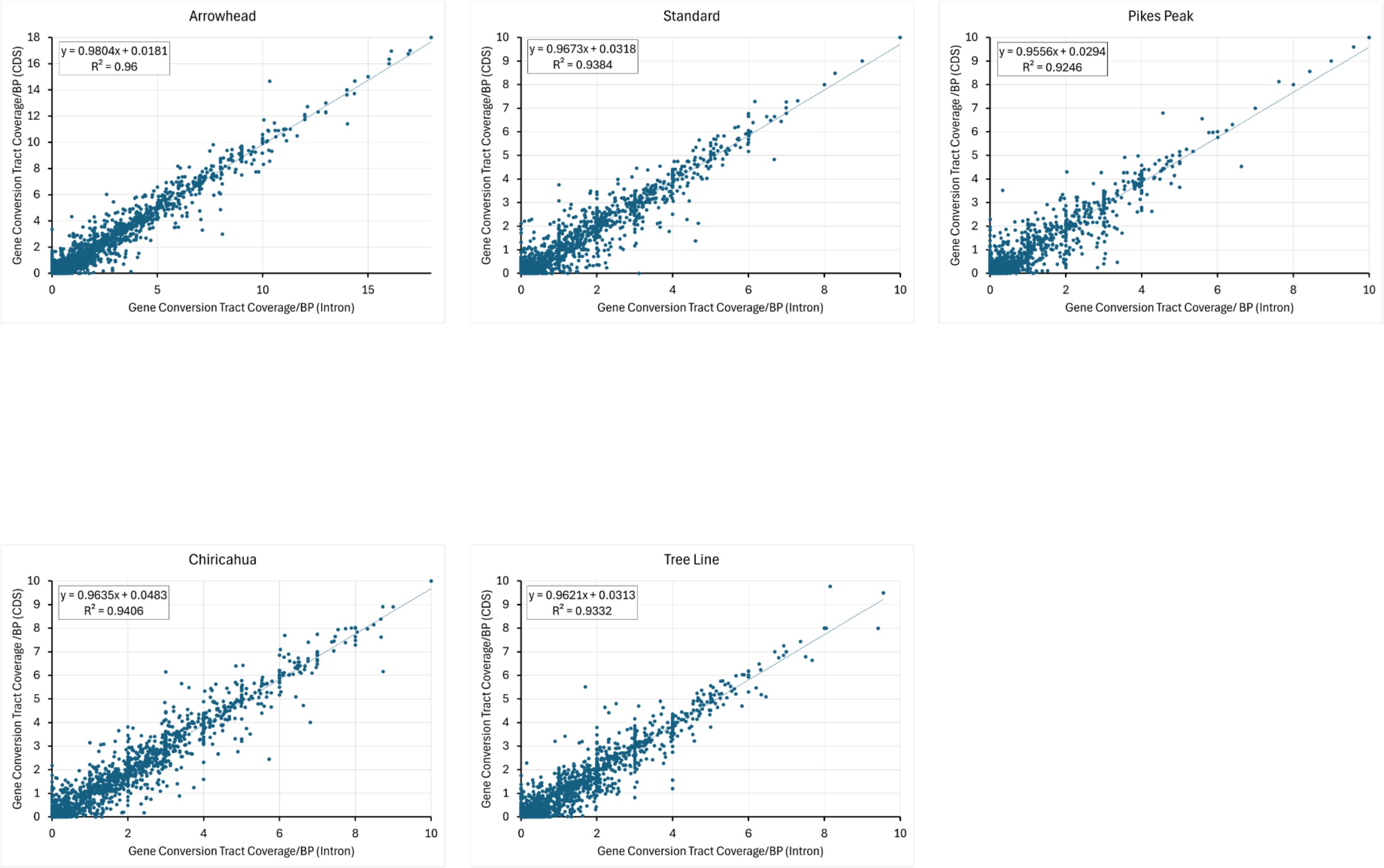


Figure S4. Plot of gene conversion tract coverage in Introns versus corresponding exons of coding sequence genes for five gene arrangements in *D. pseudoobscura*.
