# Supplementary material for: Genomics of natural populations: gene conversion events reveal selected genes within the inversions of Drosophila pseudoobscura
Source: G3 (Bethesda). 2024 Jul 29;14(10):jkae176. doi: 10.1093/g3journal/jkae176 (PMC11457094; doi:10.1093/g3journal/jkae176)
Supplement: jkae176_Supplementary_Data [file jkae176_supplementary_data.zip › Figure_S5_G3-2024-405095.pdf]

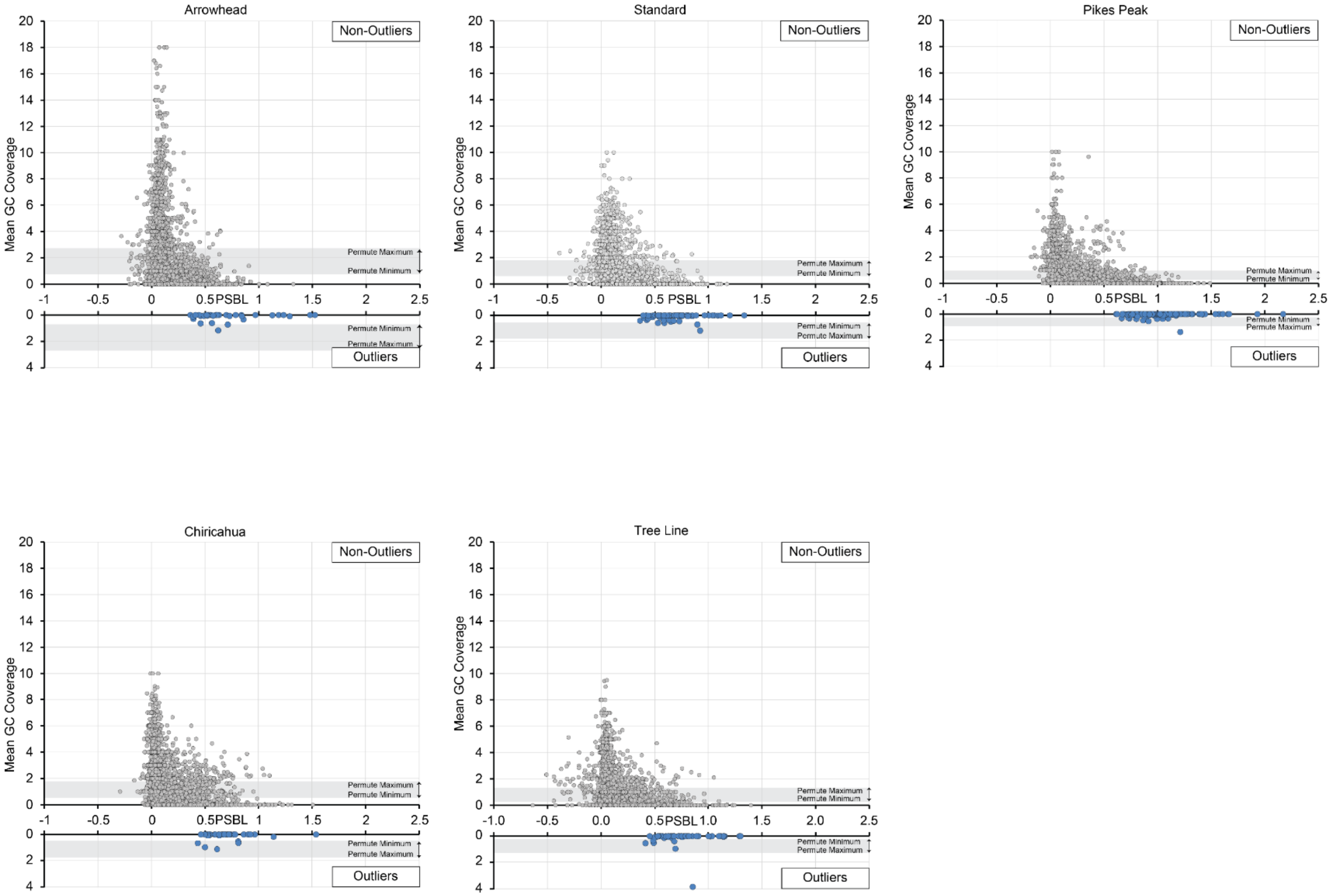


Figure S5. Relationship between Population Specific Branch Length (PSBL) on the x-axis and mean gene conversion tract coverage in 2,668 genes on Muller C of *D. pseudoobscura*. A significantly large PSBL is a proxy for a signature of selection and defines outlier genes if the gene is differentially expressed, has a fixed amino acid difference from other arrangements, or both shown as blue dots on the lower graph or gray dots on the upper graph. The gray bar highlights the minimum to maximum gene conversion coverage for genes when the gene conversion tracts within an arrangement were randomly permuted.
