# Supplementary material for: Genomics of natural populations: gene conversion events reveal selected genes within the inversions of Drosophila pseudoobscura
Source: G3 (Bethesda). 2024 Jul 29;14(10):jkae176. doi: 10.1093/g3journal/jkae176 (PMC11457094; doi:10.1093/g3journal/jkae176)
Supplement: jkae176_Supplementary_Data [file jkae176_supplementary_data.zip › Figure_S6_G3-2024-405095.pdf]

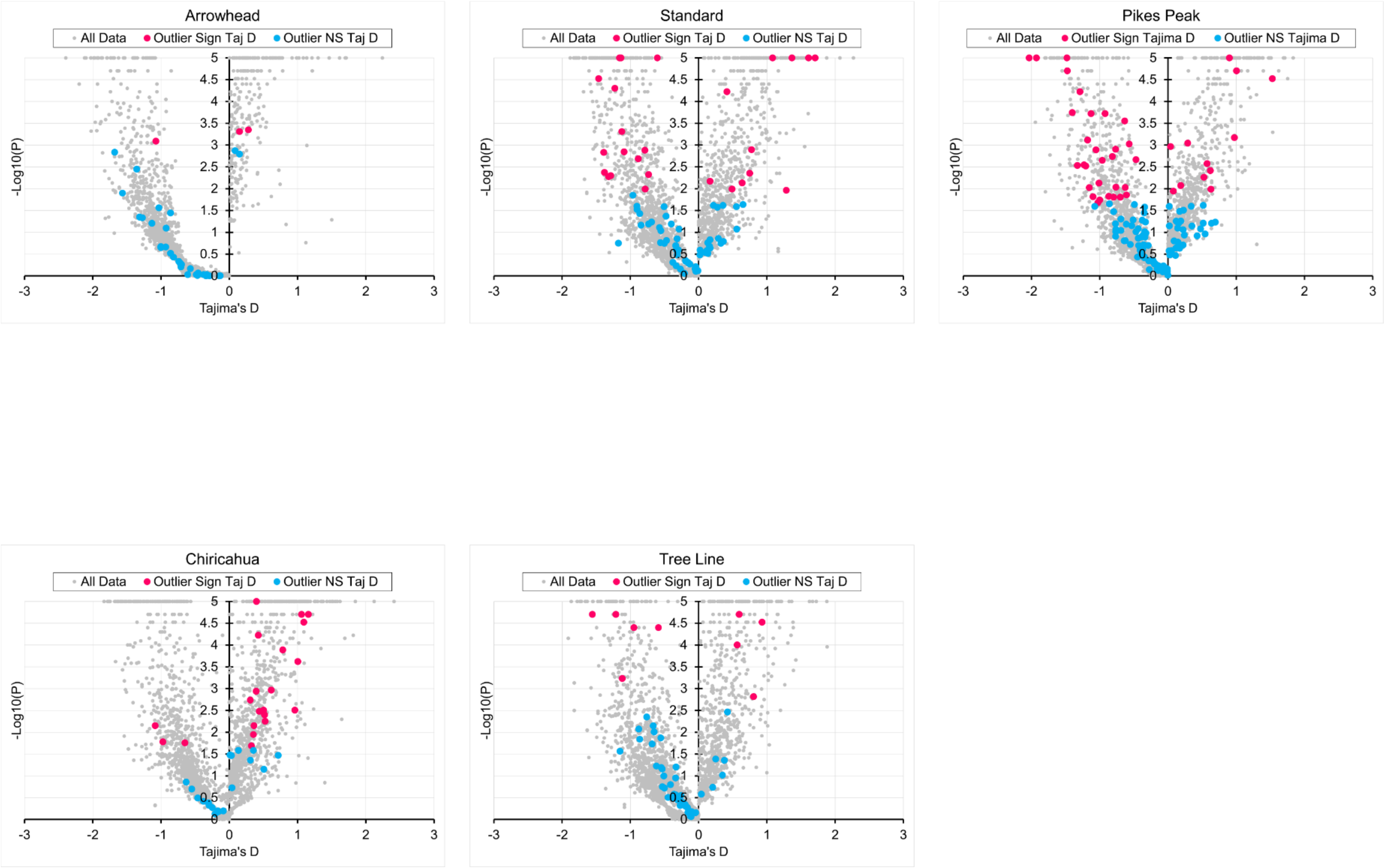

Figure S6. Plot of Tajima's D versus -Log10(P) for between 2663 to 2666 genes on Muller C of *D. pseudoobscura*. Outliers with a significantly negative or positive Tajima's *D* value are indicated with a pink dot while outliers with non-significant Tajima's *D* values are indicated with blue dots. Tajima's *D* values for non-outlier genes are indicated with gray dots.
