# Supplementary material for: Genomics of natural populations: gene conversion events reveal selected genes within the inversions of Drosophila pseudoobscura
Source: G3 (Bethesda). 2024 Jul 29;14(10):jkae176. doi: 10.1093/g3journal/jkae176 (PMC11457094; doi:10.1093/g3journal/jkae176)
Supplement: jkae176_Supplementary_Data [file jkae176_supplementary_data.zip › Table_S2_G3-2024-405095.pdf]

Table S2. Chi-Square test of homogeneity of non-outlier and outlier genes (CDSs)in different gene conversion classes for the Arrowhead gene arrangement. Gene conversion (GC) coverage classes are defined by minimum and maximum values from random permutation of gene conversion tracts.

GC Coverage Class	Non-Outlier	Outlier
**Below the Minimum**		
Coverage < 0.679		
Obs	944	35
Exp	965.8	13.2
**Between the Minimum and Maximum**		
0.679 <Coverage<2.652		
Obs	734	1
Exp	718.2	9.8
**Above Maximum**		
Coverage >2.652		
Obs	954	0
Exp	949.0	13.0
X^2 df=2	57.6	
Probability	3.09×10^{-13}	
