# Supplementary material for: Genomics of natural populations: gene conversion events reveal selected genes within the inversions of Drosophila pseudoobscura
Source: G3 (Bethesda). 2024 Jul 29;14(10):jkae176. doi: 10.1093/g3journal/jkae176 (PMC11457094; doi:10.1093/g3journal/jkae176)
Supplement: jkae176_Supplementary_Data [file jkae176_supplementary_data.zip › Table_S3_G3-2024-405095.pdf]

Table S3. Chi-Square test of homogeneity of non-outlier and outlier genes (CDSs) in different gene conversion classes for the Chiricahua gene arrangement. Gene conversion (GC) coverage classes are defined by minimum and maximum values from random permutation of gene conversion tracts.

**Coverage Class**	**Non-Outlier**	**Outlier**
**Below the Minimum**		
Coverage < 0.483		
Obs	899	37
Exp	922.0	14.0
**Between the Minimum and Maximum**		
0.483 <Coverage<1.735		
Obs	637	3
Exp	630.4	9.9
**Above the Maximum**		
Coverage >1.735		
Obs	1092	0
Exp	1075.6	16.4
X^2 df=2	59.4	
Probability	1.27×10^{-13}	
