# Supplementary material for: Genomics of natural populations: gene conversion events reveal selected genes within the inversions of Drosophila pseudoobscura
Source: G3 (Bethesda). 2024 Jul 29;14(10):jkae176. doi: 10.1093/g3journal/jkae176 (PMC11457094; doi:10.1093/g3journal/jkae176)
Supplement: jkae176_Supplementary_Data [file jkae176_supplementary_data.zip › Table_S4_G3-2024-405095.pdf]

Table S4. Chi-Square test of homogeneity of non-outlier and outlier genes (CDSs) in different gene conversion classes for the Pikes Peak gene arrangement. Gene conversion (GC) coverage classes are defined by minimum and maximum values from random permutation of gene conversion tracts.

GC Coverage Class	Non-Outlier	Outlier
**Below the Minimum**		
Coverage < 0.233		
Obs	1296	141
Exp	1356.7	80.3
**Between the Minimum and Maximum**		
0.233 <Coverage<0.922		
Obs	347	8
Exp	335.2	19.8
**Above the Maximum**		
Coverage >0.922		
Obs	876	0
Exp	827.1	48.9
X^2 df=2	108.0	
Probability	3.55×10^{-24}	
