# Supplementary material for: Genomics of natural populations: gene conversion events reveal selected genes within the inversions of Drosophila pseudoobscura
Source: G3 (Bethesda). 2024 Jul 29;14(10):jkae176. doi: 10.1093/g3journal/jkae176 (PMC11457094; doi:10.1093/g3journal/jkae176)
Supplement: jkae176_Supplementary_Data [file jkae176_supplementary_data.zip › Table_S5_G3-2024-405095.pdf]

Table S5. Chi-Square test of homogeneity of non-outlier and outlier genes (CDSs) in different gene conversion classes for the Standard gene arrangement. Gene conversion (GC) coverage classes are defined by minimum and maximum values from random permutation of gene conversion tracts.

**Coverage Class**	**Non-Outlier**	**Outlier**
**Below the Minimum**		
Coverage < 0.563		
Obs	1046	78
Exp	1089.5	34.5
**Between the Minimum and Maximum**		
0.563 <Coverage<1.758		
Obs	617	4
Exp	601.9	19.
**Above the Maximum**		
Coverage >1.758		
Obs	923	0
Exp	894.6	28.4
X^2 df=2	98.0	
Probability	5.34×10^{-22}	
