# Supplementary material for: Genomics of natural populations: gene conversion events reveal selected genes within the inversions of Drosophila pseudoobscura
Source: G3 (Bethesda). 2024 Jul 29;14(10):jkae176. doi: 10.1093/g3journal/jkae176 (PMC11457094; doi:10.1093/g3journal/jkae176)
Supplement: jkae176_Supplementary_Data [file jkae176_supplementary_data.zip › Table_S6_G3-2024-405095.pdf]

Table S6. Chi-Square test of homogeneity of non-outlier and outlier genes (CDSs) in different gene conversion classes for the Tree gene arrangement. Gene conversion (GC) coverage classes are defined by minimum and maximum values from random permutation of gene conversion tracts.

**Coverage Class**	**Non-Outlier**	**Outlier**
**Below the Minimum**<br>Coverage < 0.216<br>Obs<br>Exp	 <br>927<br>954.3	 <br>47<br>19.3
**Between the Minimum and the Maximum**<br>0.216 <Coverage<1.290<br>Obs<br>Exp	 <br>746<br>736.8	 <br>6<br>15.2
**Above the Maximum**<br>Coverage >1.290<br>Obs<br>Exp	 <br>941<br>922.9.0	 <br>1<br>19.1
X^2 df=2	61.7	
Probability	3.96×10^{-14}	
