# Supplementary figures and images for: Genomics of natural populations: gene conversion events reveal selected genes within the inversions of Drosophila pseudoobscura
Source: G3 (Bethesda). 2024 Jul 29;14(10):jkae176. doi: 10.1093/g3journal/jkae176 (PMC11457094; doi:10.1093/g3journal/jkae176)

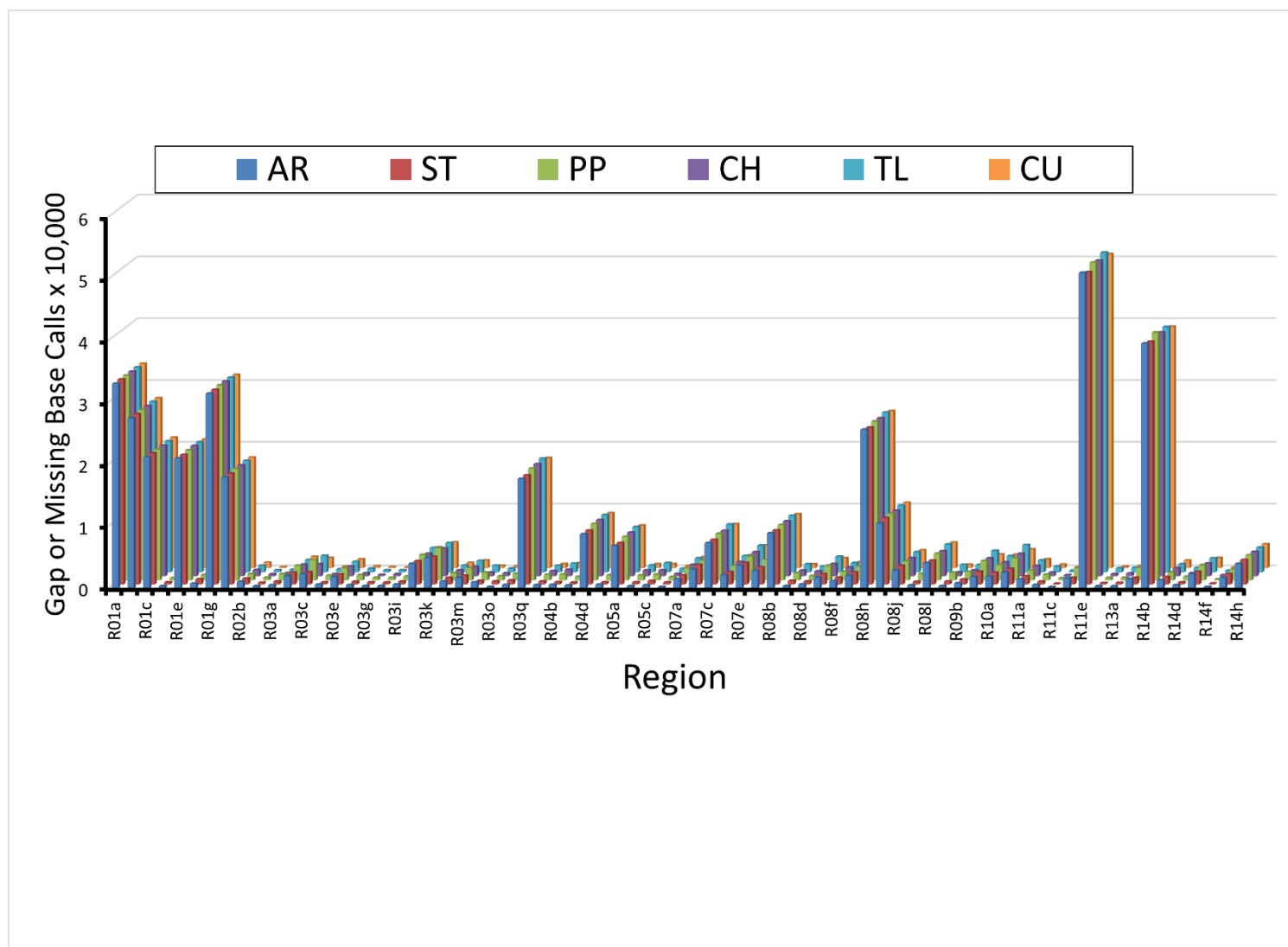

Supplement: jkae176_Supplementary_Data [file jkae176_supplementary_data.zip › Figure_S1_G3-2024-405095.pdf]
